# Urinary Oxalate Excretion and Long-Term Outcomes in Kidney Transplant Recipients

**DOI:** 10.3390/jcm8122104

**Published:** 2019-12-02

**Authors:** Alwin Tubben, Camilo G. Sotomayor, Adrian Post, Isidor Minovic, Timoer Frelink, Martin H. de Borst, M. Yusof Said, Rianne M. Douwes, Else van den Berg, Ramón Rodrigo, Stefan P. Berger, Gerjan J. Navis, Stephan J. L. Bakker

**Affiliations:** 1 Department of Internal Medicine, University Medical Center Groningen, University of Groningen, Hanzeplein 1, 9700 RB Groningen, The Netherlands; c.g.sotomayor.campos@umcg.nl (C.G.S.); a.post01@umcg.nl (A.P.); m.h.de.borst@umcg.nl (M.H.d.B.); m.y.said@umcg.nl (M.Y.S.); r.m.douwes@umcg.nl (R.M.D.); e.van.den.berg@umcg.nl (E.v.d.B.); s.p.berger@umcg.nl (S.P.B.); g.j.navis@umcg.nl (G.J.N.); s.j.l.bakker@umcg.nl (S.J.L.B.); 2 Department of Laboratory Medicine, University Medical Center Groningen, University of Groningen, Hanzeplein 1, 9700 RB Groningen, The Netherlands; i.minovic@umcg.nl; 3 Metrohm Applikon B.V., 3125 AE Schiedam, The Netherlands; timoer.frelink@metrohm.com; 4 Molecular and Clinical Pharmacology Program, Institute of Biomedical Sciences, Faculty of Medicine, University of Chile, 8380453 Santiago, Chile; rrodrigo@med.uchile.cl

**Keywords:** oxalate, hyperoxaluria, kidney transplant recipients, graft failure, post-transplantation diabetes mellitus, all-cause mortality, cardiovascular mortality, infectious mortality

## Abstract

Epidemiologic studies have linked urinary oxalate excretion to risk of chronic kidney disease (CKD) progression and end-stage renal disease. We aimed to investigate whether urinary oxalate, in stable kidney transplant recipients (KTR), is prospectively associated with risk of graft failure. In secondary analyses we evaluated the association with post-transplantation diabetes mellitus, all-cause mortality and specific causes of death. Oxalate excretion was measured in 24-h urine collection samples in a cohort of 683 KTR with a functioning allograft ≥1 year. Mean eGFR was 52 ± 20 mL/min/1.73 m^2^. Median (interquartile range) urinary oxalate excretion was 505 (347–732) µmol/24-h in women and 519 (396–736) µmol/24-h in men (*p* = 0.08), with 302 patients (44% of the study population) above normal limits (hyperoxaluria). A consistent and independent inverse association was found with all-cause mortality (HR 0.77, 95% CI 0.63–0.94, *p* = 0.01). Cause-specific survival analyses showed that this association was mainly driven by an inverse association with mortality due to infection (HR 0.56, 95% CI 0.38–0.83, *p* = 0.004), which remained materially unchanged after performing sensitivity analyses. Twenty-four-hour urinary oxalate excretion did not associate with risk of graft failure, post-transplant diabetes mellitus, cardiovascular mortality, mortality due to malignancies or mortality due to miscellaneous causes. In conclusion, in KTR, 24-h urinary oxalate excretion is elevated in 44% of KTR and inversely associated with mortality due to infectious causes.

## 1. Introduction

Kidney transplantation is considered the gold standard treatment for end-stage renal disease (ESRD) [1,2]. Short-term survival of kidney transplant recipients (KTR) has improved markedly in the past decades [3,4]. Although a better understanding of modifiable risk factors has been achieved over the recent years [5,6], patients perceive the ever existing threat of premature graft failure (GF) as most compelling, and would like to know whether factors such as lifestyle and diet can contribute to prevention of it [7,8]. Another factor of interest influencing long-term KTR survival is post-transplantation diabetes mellitus (PTDM), which has become increasingly common and may affect patient and graft survival [9]. Further, an increased risk of premature mortality, in particular, increased risk for premature death from cardiovascular and infectious causes remain significant problems in the post-transplantation setting. In KTR, conventional risk factors for cardiovascular mortality are abundantly present, such as hypertension, diabetes mellitus and dyslipidemia. On top of that, KTR had pre-existent renal diseases, which additionally increases the cardiovascular risk [10]. Mortality due to infection is significantly higher in KTR than in the general population due to multiple reasons, to which immunosuppressive therapy is a large contributing factor [11]. Furthermore, KTR are at a two to threefold higher risk of cancer-related mortality compared to the general population [12].

Although different mechanisms underlying these long-term complications of kidney transplantation have been found, substantial unknown mechanisms particular to the post-kidney transplantation setting remain to be identified in order to provide rationale for the markedly high risk of premature mortality in KTR [13]. A recent prospective cohort study in 3123 patients with chronic kidney disease (CKD) stages 2 to 4, found urinary oxalate as a potential risk factor for progression of CKD [14]. In the post-kidney transplantation setting, the study of oxalate remains overlooked. Whether urinary oxalate (reference value ≤455 µmol/24-h) [15] may be prospectively associated with adverse outcomes in KTR remains unknown. 

The current study aims to assess the potential association of urinary oxalate excretion with adverse long-term outcomes in a large cohort of extensively phenotyped KTR with a functioning graft ≥1 year. For this purpose, the prospective associations of 24-h urinary oxalate excretion with GF, PTDM, and overall and cause-specific mortality were systematically investigated.

## 2. Experimental Section

### 2.1. Study Design and Population

This is a single-center prospective cohort study, initiated in 2008 on with follow-up of endpoints until 2015. KTR with a functioning allograft for at least one year or more who visited the outpatient clinic of the University Medical Center Groningen (Groningen, The Netherlands) between November 2008 and March 2011. Exclusion criteria were no known or apparent systemic illnesses, insufficient knowledge of the Dutch language and history of drug or alcohol addiction according to their patient files. KTR received anti-hypertensive and standard maintenance immunosuppressive therapy. Of the 817 invited KTR, 706 (87%) signed informed consent. Patients missing 24-h urinary oxalate excretion were excluded from the analyses, resulting in 683 KTR eligible for statistical analyses. The study was conducted in concordance with the guidelines formulated in the Declaration of Helsinki and Istanbul, and approved by the Institutional Review Board of the UMCG (METc 2008/186) [16]. The continuous surveillance system according to the American Society of Transplantation was followed for the correct collection of data [17]. When status of patients was unknown, the referring nephrologist or general practitioners were contacted in order to obtain the missing information. There was no loss due to follow-up. 

### 2.2. Study Endpoints 

The primary endpoint of this study is death-censored GF. Secondary endpoints are PTDM, all-cause mortality and cause-specific mortality. GF occurrence in this study is defined as ESRD requiring re-transplantation or return to dialysis. A subject was considered to have developed PTDM when the fasting plasma glucose exceeded 7 mmol/L, the HbA1c exceeded 6.5% or use of antidiabetics after transplantation as registered in the patient database [18,19]. Among specific causes of death, we studied cardiovascular mortality, death from infection, death from malignancies, and other causes of death (miscellaneous). Cardiovascular mortality is defined as mortality caused by cardiovascular pathophysiology, coded by ICD-10 codes I10-I52. This information was obtained from linking the patient number to the database of the Central Bureau of Statistics and then, by physicians, reported mortality cause. Infectious mortality and mortality due to malignancies were defined as mortality caused by infectious causes or malignant causes. Miscellaneous causes of mortality have been defined as other causes of death besides the previously described outcomes.

### 2.3. Baseline Measurements and Definitions

At the outpatient clinic, baseline data was gathered according to a strict and detailed protocol described previously [20]. Anthropometrics were obtained without shoes and heavy garments. Systolic and diastolic blood pressures (SBP and DBP) were measured by means of an automatic device (Philips Suresign VS2+, Andover, MA, USA) according to a standard clinical protocol [16]. Mean arterial pressure (MAP) was automatically calculated by (SBP + DBP × 2)/3. History of cardiovascular disease was searched for in the patient files under ICD-10 code Z86.7.

Body mass index (BMI) was calculated as weight in kilograms divided by height in meters squared (kg/m^2^) [21]. Estimated glomerular filtration rate (eGFR) was calculated using the CKD Epidemiology Collaboration (CKD-EPI) creatinine equation as shown in Formula (S1) [22].

### 2.4. Assesments of Physical Activity and Dietary Intake

Physical activity was quantified using the Short Questionnaire to Assess Health-enhancing physical activity (SQUASH). Activity was expressed in intensity multiplied by the amount of hours [23]. Dietary intake was assessed using a semi-quantitative Food Frequency Questionnaire (FFQ) [24,25]. To obtain the energy of a certain product, the Dutch Food Composition Table of 2016 was used [26]. Micro and macronutrients were adjusted for total energy intake (kCal), because of the potential of correlation and confounding [27].

### 2.5. Laboratory Measurements

For the collection of 24-h urine samples, the patients were asked to start the collection the day prior to their visit to the outpatient clinic. Collection was done in concordance with a strict protocol, i.e., discarding the first morning urine, collecting the subsequent in 24 h including the next morning’s urine [16]. Subsequently, urine samples for oxalate analysis were acidified and stored at −80 °C. Urine oxalate analysis was performed using a validated ion-exchange chromatography assay with conductivity detection (Metrohm, Herisau, Switzerland). Inter-assay precision was monitored using three urine pool samples. Inter-assay precision was 8.2% at 0.17 mmol/L, 7.0% at 0.38 mmol/L and 9.0% at 0.52 mmol/L. Comparison of this method with a routine laboratory GC-MS method showed no systemic difference and no proportional difference. Reverse-phase high performance liquid chromatography (HPLC) was used to measure urinary thiosulfate [28].

### 2.6. Statistical Analyses

Data was analyzed using IBM SPSS version 23.0 (SPSS Inc., Chicago, IL, USA); Stata version 14.0 (StataCorp., College Station, TX, USA), GraphPad Prism version 7.02 (GraphPad Software, La Jolla, CA, USA), and Rstudio version 3.2.3 (R Foundation for Statistical Computing, Vienna, Austria). A two-sided *p* < 0.05 was considered significant in all following analyses. 

Normally distributed variables are expressed as mean ± standard deviation (SD), skewed data as medians (Interquartile range (IQR)), and categorical data as given number and percentage. Baseline characteristics were described for the overall population and by sex-stratified tertiles of 24-h urinary oxalate excretion. Data are presented in tertiles to allow for assessment of linearity of cross-sectional associations of 24-h urinary oxalate excretion with other variables. Sex-stratified tertiles were created by first separately distributing all female subjects according to tertiles and distributing all male subjects according to tertiles, and thereafter combining the tertiles of females and males. We generated sex-specific tertiles because of differences between women and men in oxalate excretion [29,30,31,32]. Analyses of difference in baseline characteristics across sex-stratified tertiles of 24-h urinary oxalate excretion were tested by ANOVA for normally distributed continuous variables, Kruskal-Wallis for skewed continuous variables and χ2 test for categorical data. Sex-stratified tertiles of 24-h oxalate excretion were tested for associations with outcomes by Kaplan-Meier analysis, including the log-rank test. 

Linear regression analyses were performed to investigate the association of baseline characteristics with 24-h urinary oxalate excretion. Normality was assessed by means of a *p*–*p* plot, and a natural log transformation was performed when appropriate. Homoscedasticity was controlled in a scatterplot.

Cox regression analyses were used to investigate the association of 24-h urinary oxalate with primary and secondary outcomes. Model 1 of the Cox proportional-hazards regression analysis was adjusted for demographics, i.e., sex and age. Model 2 was additionally adjusted for transplantation related variables, namely primary renal disease, BMI, donor age, time from transplantation to follow-up, eGFR and proteinuria. In the next models, baseline characteristics which were cross-sectionally associated with 24-h urinary oxalate excretion were subsequently included, and potential confounding of urinary thiosulfate was investigated due to its role in the anion transporters in the proximal renal tubuli (Model 3) [33]. In addition, we also looked for lactate dehydrogenase (LDH) because of its importance in the conversion of glyoxylate (Model 4) [34], for 24-h urinary pH because of its influence on the reaction of oxalate with calcium (Model 5) [35], for fibroblast growth factor 23 (FGF23) because of the relationship with gastrointestinal calcium absorption and oxalate bioavailability [36,37] (Model 6), and for fruits and vegetables as main dietary sources of oxalate [38,39,40] (Model 7). To allow for detection of a potential threshold effect, which was found in an earlier study on urinary oxalate excretion and CKD [14], Cox regression analyses were also performed according to sex-stratified tertiles with the first tertile as reference. 

Spline regression were created to visualize the association of 24-h urinary oxalate excretion for outcomes, for which we consistently found significant associations. Nonlinearity was tested by using the likelihood ratio test, comparing models with linear or linear and cubic spline terms. Restricted cubic splines were knotted at the minimum, median and maximum. The splines were adjusted according to Model 6 of the primary prospective analyses. 

#### Sensitivity Analyses

Several sensitivity analyses were performed to examine the robustness of the associations between 24-h urinary oxalate excretion and outcomes. For that purpose, we reanalyzed the data excluding subjects with potential inadequate 24-h urine collection (i.e., overcollection or undercollection), which was defined as the upper and lower 2.5% of the difference between the estimated and measured volume of a subject’s 24-h urine sample. The following formula was used to calculate the estimated 24-h urine volume: ¼ ((urine creatinine) * (24-h urine volume)/(serum creatinine)), where creatinine clearance was estimated using the Cockcroft-Gault Formula [41,42]. These analyses were analogous to Model 6 of the primary prospective analyses.

Furthermore, we performed competing risk analyses of outcomes of interest with all-cause mortality as competing event according to Fine and Gray [43]. For that purpose, we performed multivariable Cox regression analyses analogously to Model 6 of the primary prospective analyses. 

## 3. Results

### 3.1. Baseline Characteristics

In total 683 KTR were included in the analyses (mean age 53 ± 13, 43% female, 99.6% Caucasian ethnicity). Median urinary oxalate excretion was 505 (IQR, 347–732) µmol/24-h in women and 519 (IQR, 396–736) µmol/24-h in men (*p* = 0.08). Forty-four percent of the patients were above the range of clinical hyperoxaluria of ≤455 µmol/24-h. All 227 study subjects in tertile 3 were above the clinical cutoff point for hyperoxaluria, and all 227 subjects in tertile 1 were below the clinical cutoff point. Mean eGFR was 52 ± 20 mL/min/1.73 m^2^. Additional baseline characteristics and analyses are shown overall and by sex-stratified tertiles of 24-h urinary oxalate excretion in Table 1. 

### 3.2. Cross-Sectional Analysis

We found that age (*p* = 0.04), current smoking status (*p* = 0.01), and cystatin C (*p* = 0.03) were inversely associated with 24-h urinary oxalate excretion, whereas plasma glucose (*p* = 0.01), ascorbic acid (*p* < 0.001), fruit consumption (*p* < 0.001), vitamin B6 (*p* < 0.001), urinary urea nitrogen excretion (*p* < 0.001), and phosphate excretion (*p* < 0.001) were positively associated with 24-h urinary oxalate excretion.

### 3.3. Prospective Analyses

GF and mortality were recorded during a follow-up of 5.3 years (IQR, 4.5–6.0). During follow-up, 83 (12%) patients developed GF, 55 (9%) patients developed PTDM and 149 (22%) patients died, of which 59 deaths (40%) were due to cardiovascular causes, 41 deaths (28%) due to infectious causes, 26 deaths (17%) due to malignancies and 23 deaths (15%) due to miscellaneous causes (Table 2).

#### 3.3.1. GF, PTDM, Cardiovascular Mortality, Mortality due to Malignancies, and Miscellaneous Mortality

**Figure 1:**
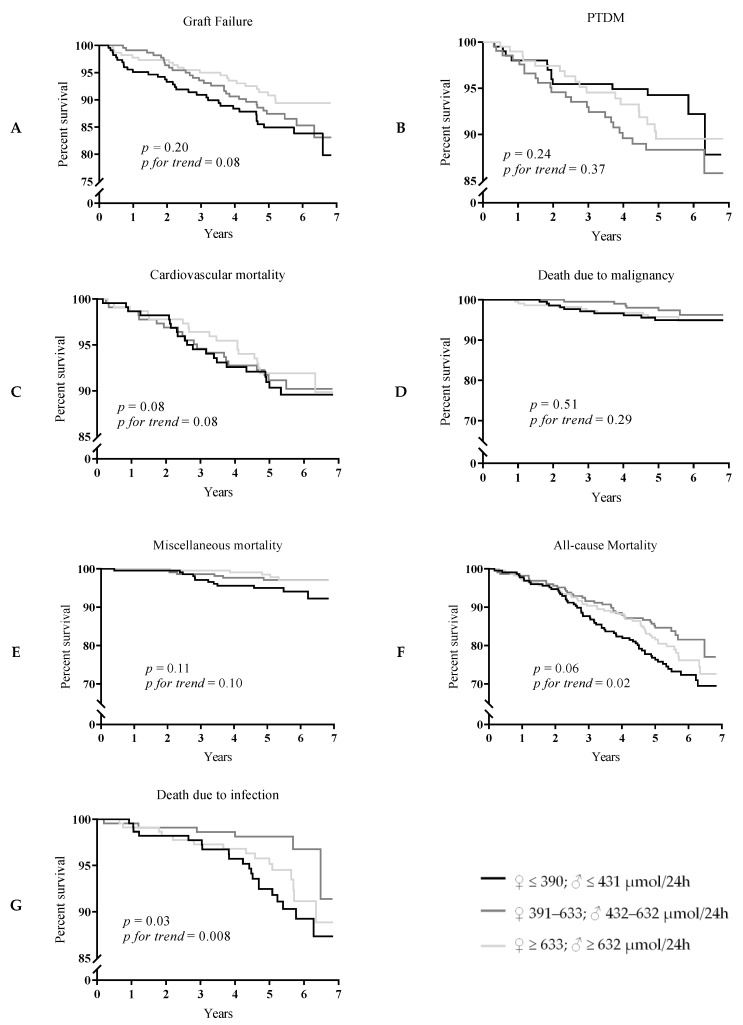
(**A**) Graft failure, (**B**) PTDM, (**C**) cardiovascular mortality, (**D**) death due to malignancy, (**E**) miscellaneous mortality (**F**) all-cause mortality, and (**G**) death due to infection according to sex-stratified tertiles of 24-hour urinary oxalate excretion over approximately 7 years of follow-up.

A Kaplan-Meier curve for the association of tertiles of 24-h urinary oxalate excretion with GF is shown in Figure 1A (*p* = 0.20, *p* for trend 0.08). Results of multivariate Cox regression analyses did not show a consistent association of 24-h urinary oxalate excretion with GF (HR 0.71, 95% CI 0.53–0.98) (Table 3). Uni- and multivariate analyses of the associations of 24-h urinary oxalate excretion and potential confounders with GF are shown in Table S1.

A Kaplan-Meier curve for the association of tertiles of 24-h urinary oxalate excretion with PTDM is shown in Figure 1B (*p* = 0.24, *p* for trend 0.37). Results of multivariate Cox regression analyses showed no association of 24-h urinary oxalate excretion with PTDM (HR 0.95, 95% CI 0.71–1.27) (Table 3). 

A Kaplan-Meier curve for the association of tertiles of 24-h urinary oxalate excretion with cardiovascular mortality is shown in Figure 1C (*p* = 0.08, *p* for trend 0.08). Results of multivariate Cox regression analyses showed cardiovascular mortality is not associated with 24-h urinary oxalate excretion (HR 0.78, 95% CI 0.56–1.10) (Table 4).

A Kaplan-Meier curve for the association of tertiles of 24-h urinary oxalate excretion with death due to malignancy is shown in Figure 1D (*p* = 0.51, *p* for trend 0.29). Results of multivariate Cox regression analyses showed mortality due to malignancies is not associated with 24-h urinary oxalate excretion (HR 1.14, 95% CI 0.73–1.77) (Table 5).

A Kaplan-Meier curve for the association of tertiles of 24-h urinary oxalate excretion miscellaneous mortality is shown in Figure 1E (*p* = 0.11, *p* for trend 0.10). Results of multivariate Cox regression analyses showed miscellaneous death causes are not associated with 24-h urinary oxalate excretion (HR 0.75, 95% CI 0.45–1.26) (Table 5).

#### 3.3.2. All-Cause and Infectious Mortality

**Figure 2:**
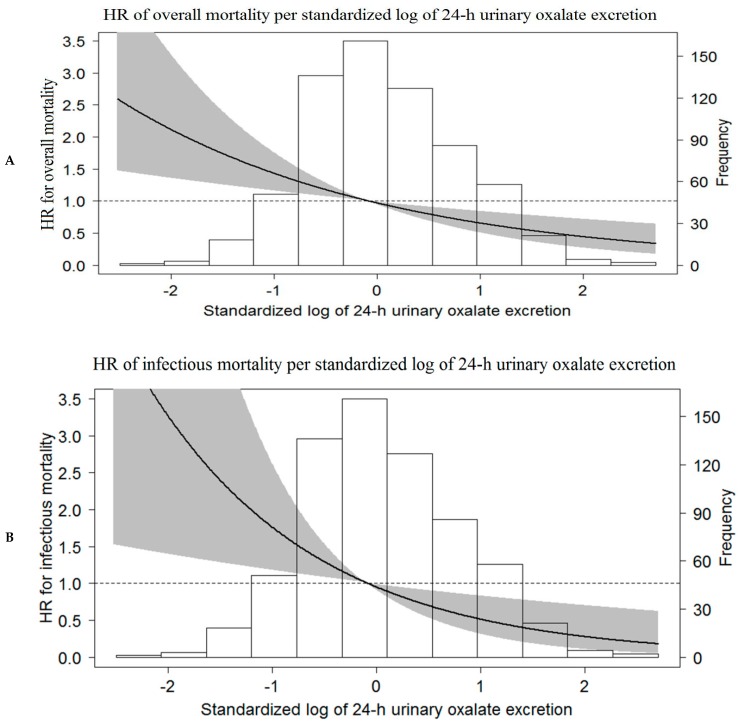
Adjusted association of standardized log 24–hour urinary oxalate excretion with (**A**) all-cause mortality, and (**B**) infectious mortality, based on restricted cubic spline regression, fitted with Model 6. The black line in the graph represents the HR, 95% CI is shown and the gray area.

A Kaplan-Meier curve for the association of tertiles of 24-h urinary oxalate excretion with all-cause mortality is shown in Figure 1F (*p* = 0.06, *p* for trend 0.02). Results of multivariate Cox regression analyses showed, however, that all-cause mortality is independently associated with 24-h urinary oxalate excretion (HR 0.77, 95% CI 0.63–0.94) (Table 4). Uni- and multivariate analyses of the associations of 24-h urinary oxalate excretion and potential confounders with all-cause mortality are shown in Table S2. The association of 24-h urinary oxalate excretion with all-cause mortality demonstrated a nonlinear relationship, as shown by a restricted cubic spline (Figure 2A).

A Kaplan-Meier curve for the association of tertiles of 24-h urinary oxalate excretion with infectious mortality is shown in Figure 1G (*p* = 0.03, *p* for trend 0.008). Results of multivariate Cox regression analyses showed infectious mortality was independently associated with 24-h urinary oxalate excretion (HR 0.58, 95% CI 0.38–0.83) (Table 5). The association between 24-h urinary oxalate excretion and infectious mortality demonstrated a nonlinear relationship, as shown by a restricted cubic spline (Figure 2B).

### 3.4. Sensitivity Analyses

When we restricted the analyses to subjects with no potential over or undercollection of 24-h urine samples based on differences in expected and observed 24-h urinary creatinine excretions (n = 650), generally similar results were found for GF (HR 0.74, 95% CI 0.55–0.99), PTDM (HR 0.93, 95% CI 0.69–1.26), cardiovascular mortality (HR 0.68, 95% CI 0.47–0.98), mortality due to malignancies (HR 1.10, 95% CI 0.70–1.72), mortality due to miscellaneous causes (HR 0.71, 95% CI 0.41–1.24), all-cause mortality (HR 0.74; 95% CI 0.59–0.92), and infectious mortality (HR 0.58, 95% CI 0.37–0.89).

When competing risk analyses were performed, generally similar results were found for PTDM (HR 1.15, 95% CI 0.91–1.48), cardiovascular mortality (HR 0.82, 95% CI 0.55–1.23), mortality due to malignancies (HR 1.16, 95% CI 0.69–1.95), mortality due to miscellaneous causes (HR 0.84, 95% CI 0.54–1.31), and infectious mortality (HR 0.61, 95% CI 0.45–0.85). The risk of GF was not consistently significant (HR 1.14, 95% CI 0.64–2.26).

## 4. Discussion

In KTR, median excretion of 24-h oxalate was higher than the clinical cut-off point for hyperoxaluria. No association of 24-h urinary oxalate excretion was found with GF, PTDM, cardiovascular mortality, or mortality due to malignancy or miscellaneous causes, but an independent, inverse association with all-cause mortality and infectious mortality was found. There was respectively a 23% and 44% decrease in hazard ratio per standard deviation increase of 24-h urinary oxalate excretion. The associations remained materially unchanged after adjusting for potential confounders. The association with all-cause and infectious mortality remained materially unchanged after performing sensitivity analyses. 

A single-centered prospective study had previously already found an elevated plasma oxalate level in KTR [44]. However, no previous study has provided data on oxalate excretion. The elevated urinary oxalate excretion reflects one of the major findings of this study, being that 44% of the stable KTR are within the clinical range of hyperoxaluria.

To the best of our knowledge, there have not been any previous studies investigating the association of urinary oxalate with GF, PTDM and (cause-specific) mortality in stable KTR. However, a recent study of Waikar et al. with CKD patients stage 2 to 4 found 24-h urinary oxalate excretion to be positively associated with all-cause mortality [14]. With regards to the study of Waikar et al., their first four quintiles can be considered to be below the range of hyperoxaluria of 455µmol/24-h, whereas in our population, only the first tertile can be considered normal with regard to urinary oxalate excretion. This, in part, might explain the difference in association of urinary oxalate excretion with the outcome variables.

The difference between Waikar et al.’s and our study cannot be explained by a higher BMI or diabetes contributing to hyperoxaluria through higher effective renal plasma flow and glomerular hyperfiltration in the Waikar et al. population (respectively, BMI of 32.1 ± 7.7 and 26.6 ± 4.8 and diabetes in 48.9% and 24% of the population) [45]. Low density lipoprotein (LDL) profile was not published in the Waikar et al. report, therefore, difference in oxalate excretion through dyslipidemia cannot be determined [46]. In both studies, the urinary samples were stored at −80 °C. Storage at this temperature can lead to underestimation of oxalate levels through calcium oxalate precipitation [14]. Since the difference of storage time of the samples until measurement is not known, we cannot exclude this as a potential clarification of the found difference. Additionally, spontaneous oxalate generation over the course of the storage might have increased the sample oxalate levels in either studies. Another hypothesis for the interesting difference in 24-h urinary oxalate excretion between the study of Waikar et al. might be found in the possible absence of *Oxalobacter formigenes* in the gut microbiome. KTR have been exposed to antimicrobial prophylactic therapies to lower the risk of opportunistic infections. This greatly affects the diversity of the human microbiome and can cause dysbiosis [47]. Dysbiosis in KTR could contribute to a decrease of *O. formigenes* and therefore, increased gastrointestinal absorption of oxalate, leading to an increased oxalate serum concentration and consequently, elevated urinary excretion. 

We found no association with GF, PTDM, mortality due to malignancies, nor mortality due to miscellaneous causes. The results of the proportional hazards models show that the inverse overall association with mortality is mainly driven by infectious mortality. We hypothesized that because 24-h urinary oxalate excretion was positively associated with ascorbic acid, which is inversely associated with overall mortality in RTR through reducing inflammation, an increase in oxalate might contribute to a lower infectious mortality [48]. However, the exact mechanism behind the association of 24-h urinary oxalate excretion with infectious mortality remains to be further investigated, since to our knowledge, there are no studies available showing a potential theoretical explanation.

The strength of this study lays in its prospective design, with a large cohort of stable KTR who were closely monitored according to standardized protocols and continuous surveillance system according to the American Society of Transplantation without loss due to follow-up during a median follow-up of 5.4 years for (specific cause) mortality. The KTR were extensively phenotyped at baseline measurement, providing a broad array of potential confounders to adjust for. The inclusion of the FFQ gives the possibility to assess the associations with dietary intake, rather than just the urinary excretion. Furthermore, urine was collected as 24-h collecting samples, according to a previously described strict protocol, which eliminates possible daily variances in fluid balance and excretion to give a more accurate excretion estimate. Additionally, potential over- or undercollection of the 24-h urine samples was accounted for by means of sensitivity analyses, which showed that the results remained materially unchanged after restricting the study population as described previously. 

However, we also acknowledge limitations of the current study. First, we were unable to adjust our results for socioeconomic status at baseline. Next, although the FFQ and SQUASH are validated questionnaires, they are self-reported, which may lead to possible over or underreporting of dietary intake and physical activity. We also acknowledge that our population consists almost entirely of Caucasian ethnicity, therefore, our results call for caution to extrapolate our results to different populations with regard to ethnicity. Finally, data on nephrolithiasis was not documented; therefore, we were unable to assess the association of urinary oxalate with the outcome nephrolithiasis, which remains a rather overlooked topic in KTR. Nevertheless, our results show for the first time a high prevalence of hyperoxaluria in the post-kidney transplant setting, thus emphasizing the need for future studies in which such analyses are performed. Additionally, because the study of the microbiome was beyond the scope of the current study, the hypothesized mechanism of increased gastrointestinal absorption of oxalate to explain the observed levels of hyperoxaluria cannot be further confirmed.

In conclusion, in stable KTR, 24-h urinary oxalate excretion is quantitatively higher than in the general population. Forty-four percent of the current study population showed urinary oxalate levels above the range of clinical hyperoxaluria. This hyperoxaluria might suggest a role of dysbiosis by leading to diminished *O. formigenes* and therefore, higher oxalate absorption and excretion in the current study population. Twenty-four-hour urinary oxalate excretion was not associated with risk of graft failure, post-transplant diabetes mellitus, cardiovascular mortality, mortality due to malignancies, nor death from miscellaneous causes. However, a consistent and independent inverse association was found with infectious mortality. Our data encourages further studies to validate our findings on the associations of oxalate with long-term outcomes in KTR. Future studies are warranted to investigate specific causes of death and the effect of hyperoxaluria post-kidney transplantation. 

## Figures and Tables

**Table 1:** Baseline characteristics of the overall population, and by sex-stratified tertiles of 24-h urinary oxalate excretion. ^a^

Baseline Characteristics	Overall KTR *n* = 683	Sex-Stratified Groups of 24-h Urinary Oxalate Excretion			*p*
		♀ ≤ 390; ♂ ≤ 431	♀ 391–633; ♂ 432–632	♀ ≥ 633; ♂ ≥ 632	
		µmol/24-h	µmol/24-h	µmol/24-h	
**Oxalate**					
Oxalate in 24-h urine, µmol ^b^	514 (378–732)	339 (278–278)	514 (461–563)	882 (732–1137)	—
**Demographics**					
Age, years	53 ± 13	54 ± 13	53 ± 12	51 ± 13	0.04
Sex (female), *n* (%)	295 (43)	98 (43)	99 (43)	98 (43)	1.00
Ethnicity (Caucasian), *n* (%)	680 (99.6)	226 (99.6)	228 (99.6)	226 (99.6)	1.00
**Body composition**					
BSA, m^2^	1.94 ± 0.22	1.92 ± 0.21	1.98 ± 0.21	1.94 ± 0.23	0.05
BMI, kg/m^2^	26.6 ± 4.8	26.3 ± 4.8	27.0 ± 4.6	26.6 ± 4.8	0.34
Waist circumference, cm	98 (89–108)	97 (89–105)	100 (90–110)	96 (87–106)	0.02
**Lifestyle**					
Current smoker, *n* (%)	81 (12)	42 (19)	19 (9)	20 (9)	0.001
Alcohol consumption					0.30
None, *n* (%)	22 (3)	6 (3)	8 (4)	8 (4)	
0–10 g/day, *n* (%)	426 (62)	144 (64)	146 (64)	136 (60)	
10–30 g/day, *n* (%)	137 (20)	44 (19)	44 (19)	49 (22)	
>30 g/dag, *n* (%)	37 (5)	10 (4)	12 (5)	15 (7)	
SQUASH-score	5070 (2040–7800)	4440 (1680–7240)	5400 (2323–8475)	5580 (2280–7980)	0.10
**Cardiovascular**					
History of CV disease, *n* (%)	295 (50)	92 (41)	103 (45)	100 (44)	0.72
SBP, mmHg	136 ± 18	137 ± 17	136 ± 18	135 ± 18	0.42
MAP, mmHg (calculated)	100 ± 12	101 ± 11	100 ± 12	100 ± 13	0.73
LDL cholesterol, mmol/L	3.0 ± 0.9	3.1 ± 0.9	3.0 ± 0.9	2.9 ± 0.9	0.54
Triglycerides, mmol/L	1.7 (1.2–2.3)	1.7 (1.3–2.3)	1.6 (1.2–2.4)	1.7 (1.2–2.2)	0.69
**Glucose homeostasis**					
Diabetes mellitus, *n* (%)	162 (24)	52 (23)	58 (25)	53 (23)	0.78
Plasma glucose, mmol/L	5.3 (4.8–6.0)	5.3 (4.8–5.9)	5.2 (4.8–6.2)	5.3 (4.7–6.1)	0.85
**Diet**					
Av. energy intake, kCal/day	2092 (1720–2536)	2045 (1705–2479)	2104 (1735–2557)	2171 (1759–2589)	0.45
Av. daily fat intake, g/d ^c^	84 (65–106)	80 (63–101)	85 (66–106)	86 (64–110)	0.31
Av. daily protein intake, g/d ^c^	81 (67–95)	80 (65–95)	81 (66–95)	81 (68–95)	0.82
Glycine, mg/d ^c^	3276 ± 806	3228 ± 805	3261 ± 817	3337 ± 794	0.38
Ascorbic acid, mg/d ^c^	84 (60–118)	70 (53–101)	82 (60–114)	103 (73–138)	<0.001
Vegetables, g/d	93 ± 58	94 ± 53	90 ± 53	96 ± 66	0.54
Fruits, g/d	123 (65–232)	111 (50–226)	121 (64–228)	165 (81–247)	0.001
**Transplantation characteristics**					
Age donor, years	43 ± 15	43 ± 15	43 ± 16	43 ± 16	0.85
Sex donor (female), *n* (%)	322 (47)	108 (49)	99 (44)	15 (52)	0.24
Donor type (living), *n* (%)	231 (34)	67 (30)	81 (35)	83 (37)	0.24
**Serum markers**					
Venous pCO2, kPa	5.9 ± 0.8	5.9 ± 0.9	5.9 ± 0.8	5.8 ± 0.8	0.53
Leukocyte count, per 10^9^/L	8.2 ± 2.7	8.3 ± 2.6	8.1 ± 2.8	8.1 ± 2.6	0.52
HsCRP, mg/L	1.6 (0.7–4.6)	1.6 (0.8–4.4)	2.0 (0.8–5.3)	1.4 (0.6–3.8)	0.04
Hemoglobin, mmol/L	8.2 ± 1.1	8.1 ± 1.1	8.2 ± 1.1	8.3 ± 1.1	0.11
FGF-23	61 (43–99)	63 (43–107)	61 (42–98)	61 (45–97)	0.66
LDH, U/L	198 (170–232)	195 (169–232)	203 (174–238)	196 (170–223)	0.35
Vitamin B6, nmol/L	29 (18–29)	27 (16–47)	26 (15–44)	36 (22–57)	<0.001
**Renal allograft function**					
Creatinine, µmol/L	125 (100–160)	126 (99–164)	126 (101–164)	122 (100–157)	0.66
eGFR, mL/min/1.73 m^2^	52 ± 20	51 ± 20	52 ± 20	54 ± 20	0.26
Serum cystatin C, mg/L	1.7 (1.3–2.2)	1.7 (1.3–2.5)	1.7 (1.3–2.2)	1.6 (1.3–2.1)	0.25
Proteinuria ≥ 0.5 g/24-h, *n* (%)	152 (22)	49 (22)	49 (21)	54 (24)	0.59
**24-h urine**					
pH	6.0 ± 0.5	6.0 ± 0.5	6.0 ± 0.5	6.1 ± 0.5	0.48
UUN excretion, mmol	389 ± 114	349 ± 100	407 ± 111	412 ± 1	<0.001
Phosphate excretion, mmol	25 ± 9	22 ± 8	25 ± 8	27 ± 9	<0.001
Thiosulfate excretion, µmol	7.0 (3.9–12.0)	6.7 (3.7–11.0)	6.9 (4.2–12.5)	7.5 (3.8–12.6)	0.57
Protein excretion, mg	196 (15–367)	163 (15–281)	221 (15–380)	200 (15–417)	0.10

Abbreviations: ♀, female; ♂, male; KTR, kidney transplant recipients; *n*, number; β, standardized beta; BSA, body surface area; BMI, body mass index; SQUASH, Short Questionnaire to Assess Health-enhancing physical activity; SBP, systolic blood pressure; MAP, mean arterial pressure; LDL, low density lipoprotein; Av., average; hs-CRP, high sensitivity C-reactive protein; LDH, lactate dehydrogenase; eGFR, estimated glomerular filtration rate; UUN, urinary urea nitrogen; FGF-23, fibroblast growth factor 23. ^a^ Normally distributed variables are expressed as mean ± standard deviation (SD), skewed data as medians (25th–75th inter quartile range (IQR)), categorical data is given as number and percentage, *n,* (%). Analyses of difference in baseline characteristics across sex-stratified tertiles of 24-h urinary oxalate excretion were tested by ANOVA for normally distributed continuous variables; Kruskal-Wallis for skewed continuous variables; χ^2^ test for categorical data. ^b^ To convert oxalate in µmol/24-h to mg/24-h, multiply by 0.088. ^c^ Adjusted for energy intake.

**Table 2:** Association of baseline characteristics with 24-h urinary oxalate excretion. ^a^

Baseline Characteristics	β	*p*
**Demographics**		
Age, years	−0.08	0.04
**Lifestyle**		
Current smoker	−0.11	0.01
Plasma glucose, mmol/L	0.10	0.01
**Diet**		
Ascorbic acid, mg/d ^C^	0.24	<0.001
Fruits, g/d	0.16	<0.001
**Blood markers**		
Vitamin B6 in blood, nmol/L	0.20	<0.001
**Renal allograft function**		
Cystatin C, blood, mg/L	−0.16	0.03
**24-h Urine**		
UUN excretion, mmol	0.24	<0.001
Phosphate excretion, mmol	0.25	<0.001

^a^ Multivariate linear regression, adjusted for age, sex and eGFR.

**Table 3:** Association of 24-h urine oxalate excretion with graft failure and PTDM.

	Continuous,		Tertiles				
	per 1–SD		Tertile 1	Tertile 2		Tertile 3	
	HR	95%CI	Ref	HR	95% CI	HR	95% CI
**Graft Failure**							
Model 1	0.80	0.64–1.00	1.00	0.82	0.50–1.36	0.58	0.33–1.00
Model 2	0.78	0.61–1.02	1.00	0.77	0.45–1.32	0.61	0.35–1.08
Model 3	0.72	0.54–0.94	1.00	0.68	0.39–1.17	0.48	0.26–0.86
Model 4	0.71	0.53–0.93	1.00	0.68	0.40–1.18	0.45	0.24–0.82
Model 5	0.71	0.53–0.93	1.00	0.66	0.38–1.15	0.43	0.23–0.80
Model 6	0.71	0.53–0.98	1.00	0.69	0.39–1.20	0.44	0.24–0.83
Model 7	0.75	0.56–1.00	1.00	0.70	0.40–1.25	0.48	0.19–0.77
**PTDM**							
Model 1	0.93	0.71–1.22	1.00	1.27	0.68–2.37	0.71	0.34–1.46
Model 2	0.91	0.69–1.23	1.00	1.23	0.66–2.32	0.68	0.33–1.41
Model 3	0.91	0.68–1.22	1.00	1.32	0.70–2.50	0.61	0.28–1.33
Model 4	0.94	0.70–1.25	1.00	1.39	0.73–2.68	0.66	0.30–1.44
Model 5	0.95	0.73–1.27	1.00	1.50	0.77–2.91	0.71	0.32–1.57
Model 6	0.95	0.71–1.27	1.00	1.50	0.77–2.91	0.76	0.34–1.73
Model 7	0.99	0.73–1.33	1.00	1.45	0.74–2.83	0.75	0.34–1.69

Multivariate Cox regression were performed for the association of 24-h urinary oxalate excretion with graft failure and PTDM. Model 1: age and sex adjusted. Model 2: Model 1 + adjustment for BMI, primary renal disease, donor age, transplant vintage, eGFR, and proteinuria. Model 3: Model 2 + adjustment for thiosulfate in 24-h urine. Model 4: Model 3 + adjustment for LDH in blood. Model 5: Model 4 + adjustment for pH of 24-h urine. Model 6: Model 5 + adjustment for FGF23. Model 7: Model 6 + adjustment for fruit and vegetables intake.

**Table 4:** Association of 24-h urine oxalate excretion with all-cause and cardiovascular mortality.

	Continuous,		Tertiles				
	per 1–SD		Tertile 1	Tertile 2		Tertile 3	
	HR	95% CI	Ref	HR	95% CI	HR	95% CI
**All-cause mortality**							
Model 1	0.83	0.70–0.98	1.00	0.86	0.59–1.25	0.72	0.48–1.74
Model 2	0.81	0.67–0.97	1.00	0.84	0.57–1.23	0.73	0.48–1.14
Model 3	0.76	0.62–0.93	1.00	0.85	0.58–1.25	0.56	0.35–0.88
Model 4	0.76	0.62–0.92	1.00	0.80	0.54–1.18	0.53	0.34–0.83
Model 5	0.77	0.63–0.94	1.00	0.79	0.53–1.17	0.54	0.34–0.86
Model 6	0.77	0.63–0.94	1.00	0.75	0.50–1.13	0.55	0.34–0.86
Model 7	0.83	0.68–1.03	1.00	0.74	0.48–1.15	0.67	0.41–1.11
**Cardiovascular mortality**							
Model 1	0.90	0.69–1.19	1.00	0.90	0.48–1.69	1.09	0.59–2.00
Model 2	0.87	0.65–1.17	1.00	0.84	0.44–1.62	1.11	0.59–2.08
Model 3	0.78	0.56–1.09	1.00	0.87	0.45–1.69	0.81	0.40–1.63
Model 4	0.77	0.56–1.08	1.00	0.81	0.42–1.57	0.75	0.37–1.53
Model 5	0.79	0.57–1.09	1.00	0.82	0.42–1.59	0.78	0.38–1.57
Model 6	0.78	0.56–1.10	1.00	0.82	0.41–1.62	0.77	0.37–1.59
Model 7	0.79	0.55–1.13	1.00	0.80	0.39–1.66	0.75	0.33–1.70

Multivariate Cox regression were performed for the association of 24-h urinary oxalate excretion with all-cause and cardiovascular mortality. Model 1: age and sex adjusted. Model 2: Model 1 + adjustment for BMI, primary renal disease, donor age, transplant vintage, eGFR, and proteinuria. Model 3: Model 2 + adjustment for thiosulfate in 24-h urine. Model 4: Model 3 + adjustment for LDH in blood. Model 5: Model 4 + adjustment for pH of 24-h urine. Model 6: Model 5 + adjustment for FGF23. Model 7: Model 6 + adjustment for fruit and vegetables intake.

**Table 5:** Association of 24-h urine oxalate excretion with death due to infection, malignancy and other causes.

	Continuous,		Tertiles				
	per 1–SD		Tertile 1	Tertile 2		Tertile 3	
	HR	95% CI	Ref	HR	95% CI	HR	95% CI
**Death due to infection**							
Model 1	0.67	0.49–0.92	1.00	0.75	0.39–1.47	0.33	0.13–0.83
Model 2	0.58	0.40–0.83	1.00	0.75	0.38–1.49	0.31	0.12–0.79
Model 3	0.54	0.36–0.81	1.00	0.70	0.35–1.40	0.25	0.09–0.68
Model 4	0.56	0.38–0.82	1.00	0.65	0.32–1.30	0.23	0.09–0.63
Model 5	0.57	0.38–0.84	1.00	0.65	0.32–1.32	0.24	0.09–0.66
Model 6	0.56	0.38–0.83	1.00	0.62	0.31–1.26	0.25	0.09–0.67
Model 7	0.58	0.38–0.88	1.00	0.57	0.27–1.21	0.30	0.11–0.83
**Death due to malignancy**							
Model 1	1.01	0.69–1.50	1.00	1.31	0.54–3.17	0.78	0.28–2.20
Model 2	0.98	0.65–1.47	1.00	1.31	0.54–3.18	0.74	0.26–2.09
Model 3	1.02	0.68–1.53	1.00	1.50	0.60–3.77	0.84	0.29–2.45
Model 4	1.03	0.68–1.55	1.00	1.56	0.62–3.94	0.88	0.30–2.59
Model 5	1.08	0.71–1.62	1.00	1.44	0.56–3.71	0.95	0.32–2.81
Model 6	1.10	0.71–1.71	1.00	1.24	0.45–3.41	1.01	0.33–3.07
Model 7	1.14	0.73–1.77	1.00	1.08	0.36–3.8	1.18	0.37–3.71
**Death due to other causes**							
Model 1	0.76	0.48–1.21	1.00	0.63	0.23–1.71	070	26–1.89
Model 2	0.82	0.51–1.35	1.00	0.53	0.19–1.47	0.62	0.22–1.74
Model 3	0.77	0.45–1.29	1.00	0.59	0.21–1.65	0.43	0.13–1.41
Model 4	0.76	0.45–1.27	1.00	0.53	0.19–1.51	0.39	0.12–1.29
Model 5	0.76	0.46–1.28	1.00	0.53	0.19–1.51	0.39	0.12–1.29
Model 6	0.75	0.45–1.26	1.00	0.46	0.16–1.35	0.36	0.11–1.20
Model 7	0.96	0.56–1.63	1.00	0.64	0.19–2.19	0.75	0.20–2.74

Multivariate Cox regression were performed for the association of 24-h urinary oxalate excretion with death due to infection, malignancy and other causes. Model 1: age and sex adjusted. Model 2: Model 1 + adjustment for BMI, primary renal disease, donor age, transplant vintage, eGFR, and proteinuria. Model 3: Model 2 + adjustment for thiosulfate in 24-h urine. Model 4: Model 3 + adjustment for LDH in blood. Model 5: Model 4 + adjustment for pH of 24-h urine. Model 6: Model 5 + adjustment for FGF23. Model 7: Model 6 + adjustment for fruit and vegetables intake.

## Supplementary Materials

The following are available online at https://www.mdpi.com/2077-0383/8/12/2104/s1, Formula (S1): CKD-EPI creatinine according to Levey et al., Supplementary Table S1: Uni- and multivariate analyses of the associations of 24-h urinary oxalate excretion and potential confounders with graft failure; Supplementary Table S2: Uni- and multivariate analyses of the associations of 24-h urinary oxalate excretion and potential confounders * with all-cause mortality.
