# Supplementary material for: Urinary Oxalate Excretion and Long-Term Outcomes in Kidney Transplant Recipients
Source: J Clin Med. 2019 Dec 2;8(12):2104. doi: 10.3390/jcm8122104 (PMC6947615; doi:10.3390/jcm8122104)
Supplement: Supplementary file 1 [file jcm-08-02104-s001.pdf]

Journal of
Clinical Medicine

MDPI

Article

Supplementary Material: Urinary Oxalate Excretion and Long-term Outcomes in Kidney Transplant Recipients

Alwin Tubben, BSc[1,*]; Camilo G. Sotomayor, MD[1]; Adrian Post, MSc[1]; Isidor Minovic, PhD[2]; Timoer Frelink, PhD[3]; Martin H. de Borst, MD, PhD[1]; M. Yusof Said, MD[1]; Rianne M. Douwes, MD[1]; Else van den Berg, MD, PhD[1]; Ramón Rodrigo, MSc[4]; Stefan P. Berger, MD, PhD[1]; Gerjan J. Navis, MD, PhD[1]; Stephan J.L. Bakker, MD, PhD[1]

[1] Department of Internal Medicine. University Medical Center Groningen. University of Groningen. Hanzeplein 1. Groningen 9700 RB. The Netherlands; c.g.sotomayor.campos@umcg.nl (C.G.S.); a.post01@umcg.nl (A.P.); m.h.de.borst@umcg.nl (M.H.d.B.); m.y.said@umcg.nl (M.Y.S.); r.m.douwes@umcg.nl (R.M.D.); e.van.den.berg@umcg.nl (E.v.d.B.); s.p.berger@umcg.nl (S.B.P.); g.j.navis@umcg.nl (G.J.N.); s.j.l.bakker@umcg.nl (S.J.L.B.)

[2] Department of Laboratory Medicine. University Medical Center Groningen. University of Groningen. Hanzeplein 1. Groningen 9700 RB. The Netherlands; i.minovic@umcg.nl

[3] Metrohm Applikon B.V.. 3125 AE Schiedam. the Netherlands; timoer.frelink@metrohm.com

[4] Molecular and Clinical Pharmacology Program. Institute of Biomedical Sciences. Faculty of Medicine. University of Chile. 8380453 Santiago. Chile; rrodrigo@med.uchile.cl

* Correspondence: a.tubben@umcg.nl; Tel: +31 6 380 741 93

Received: date; Accepted: date; Published: date

CKD–EPI creatinine: eGFR = 141 * min(Scr/κ. 1)$^{\alpha}$ * max(Scr/κ. 1)$^{-1.209}$ * 0.993^{Age} * 1.018 [if female] * 1.159 [if black].

Formula S1. CKD–EPI creatinine according to Levey et al. Serum creatinine (Scr) is denoted in mg/dL. κ is 0.7 for females and 0.9 for males and α is –0.329 if female and –0.411 if male. Min stands for the minimum of Scr/κ or 1. Max stands for the maximum of Scr/κ or 1.

Supplementary Table 1. Uni- and multivariate analyses of the associations of 24-hour urinary oxalate excretion and potential confounders* with graft failure

	Univariate analyses			Multivariate analyses		
	**HR**	**95% CI**	***p***	**HR**	**95% CI**	***p***
Oxalate excretion, per SD	0.81	0.65-1.02	0.07	0.71	0.53-0.94	0.02
Age, per 5 years	0.92	0.85-0.96	0.04	0.88	0.78-0.99	0.04
Sex, female	1.07	0.69-1.65	0.77	1.29	0.75-2.22	0.37
BMI, per kg/m^2	1.00	0.95-1.05	0.92	0.99	0.94-1.06	0.84
Primary kidney disease						
Glomerulonephritis	1.37	0.58-3.24	0.47	2.43	0.97-6.10	0.06
Tubulo-interstial nephritis	1.38	0.66-2.78	0.39	1.26	0.53-3.01	0.60
Cystic kidneys	1.15	0.60-2.22	0.67	1.32	0.63-2.79	0.46
Hypo- or dysplasia	1.09	0.32-3.66	0.89	0.84	0.17-4.02	0.83
Renovascular diseases	2.23	0.98-5.08	0.06	1.54	0.57-4.18	0.40
Diabetes	1.74	0.65-1.63	0.27	1.76	0.82-3.76	0.15
Miscellaneous	1.15	0.75-2.32	0.69	1.75	0.82-3.76	0.15
Age donor, per 5 years	1.02	0.95-1.10	0.58	0.97	0.90-1.10	0.93
eGFR, per 10 mL/min/1,73 m^2	0.49	0.41-0.57	<0.001	0.55	0.45-0.68	<0.001
Proteinuria, $\geq$0,5g/24-hour	7.69	4.91-12.05	<0.001	5.13	2.99-8.83	<0.001
Transplant vintage, per year	1.00	0.97-1.03	0.96	1.01	0.97-1.05	0.61
Thiosulfate excretion, per SD	0.57	0.44-0.73	<0.001	0.83	0.58-1.20	0.32
Plasma LDH, per SD	2.52	1.04-6.12	0.04	1.53	0.49-4.85	0.47
pH of 24-hour urine, per 1 unit	0.47	0.30-0.74	0.001	0.64	0.38-1.06	0.08
Plasma FGF-23, per SD	2.91	2.39-3.55	<0.001	1.68	1.21-2.35	0.002

*Potential confounders are listed according to model 6 of primary prospective analyses.

Supplementary Table 2. Uni- and multivariate analyses of the associations of 24-hour urinary oxalate excretion and potential confounders* with all-cause mortality

	Univariate analyses			Multivariate analyses		
	**HR**	**95% CI**	***p***	**HR**	**95% CI**	***p***
Oxalate excretion, per SD	0.79	0.67-0.94	0.01	0.77	0.63-0.94	0.01
Age, per 5 years	1.39	1.28-1.50	<0.001	1.41	1.27-1.56	<0.001
Sex, female	1.16	0.83-1.61	0.38	1.19	0.81-1.76	0.38
BMI, per kg/m^2	1.02	0.99-1.06	0.17	0.92	0.93-1.01	0.19
Primary kidney disease						
Glomerulonephritis	0.82	0.36-1.84	0.62	1.12	0.49-2.57	0.79
Tubulo-interstial nephritis	0.99	0.55-1.88	0.97	1.42	0.67-2.95	0.37
Cystic kidneys	1.60	1.00-2.56	0.05	1.56	0.92-2.65	0.10
Hypo- or dysplasia	1.34	0.56-3.20	0.51	4.23	1.58-11.32	0.01
Renovascular diseases	1.48	0.71-3.10	0.30	0.80	0.32-2.00	0.64
Diabetes	4.01	2.28-7.05	<0.001	3.68	1.93-7.01	<0.001
Miscellaneous	1.37	0.81-2.30	0.24	1.37	0.78-2.42	0.28
Age donor, per 5 years	1.01	0.96-1.06	0.75	0.80	0.92-1.06	0.99
eGFR, per 10 mL/min/1,73 m^2	1.59	1.33-1.90	<0.001	0.93	0.84-1.04	0.22
Proteinuria, ≥0,5g/24-hour	2.03	1.45-2.84	<0.001	2.18	1.45-3.27	<0.001
Transplant vintage, per year	1.02	1.00-1.04	0.14	1.00	0.97-1.03	0.93
Thiosulfate excretion, per SD	0.72	0.59-0.88	0.02	0.69	0.54-0.88	0.01
Plasma LDH, per SD	4.66	2.48-8.76	<0.001	3.29	1.52-7.13	0.01
pH of 24-hour urine, per 1 unit	0.82	0.59-1.13	0.22	0.69	0.47-1.00	0.05
Plasma FGF-23, per SD	1.59	1.33-1.90	<0.001	1.27	0.95-1.71	0.11

*Potential confounders are listed according to model 6 of primary prospective analyses.
